# Limbal niche cells can reduce the angiogenic potential of cultivated oral mucosal epithelial cells

**DOI:** 10.1186/s11658-018-0133-x

**Published:** 2019-04-04

**Authors:** Chao-Ye Duan, Hua-Tao Xie, Xin-Yue Zhao, Wen-Han Xu, Ming-Chang Zhang

**Affiliations:** Department of Ophthalmology, Union Hospital, Tongji Medical College, Huazhong University of Science and Technology, Wuhan, 430022 China

**Keywords:** Limbal niche cells, 3T3 cells, Limbal stem cell deficiency, Oral mucosal epithelial transplantation, Corneal neovascularization

## Abstract

**Background:**

Autologous cultivated oral mucosal epithelial transplantation (COMET) is an important treatment for limbal stem cell deficiency. However, peripheral corneal neovascularization after surgery hinders its application. This study aims to employ a culture system using allogenic limbal niche cells (LNCs) instead of mouse-derived 3T3 cells as a feeder layer that could relieve postoperative neovascularization.

**Methods:**

Rat oral mucosal epithelial cells (OMECs) were co-cultured with rat LNCs or 3T3 cells. Cultivated oral mucosal epithelial cells (COMECs) of different culture systems were identified by hematoxylin and eosin staining and immunocytochemistry. The expression levels of the angiogenesis-related factors were analyzed by RT-qPCR and western blotting/ELISA. Angiogenic potential was reconfirmed by cell viability and tube formation assays with human umbilical vein endothelial cells (HUVECs).

**Results:**

COMECs were obtained from both culture systems successfully. Immunocytochemistry showed approximately equal percentages of positive staining cells for p63α (*p* = 0.9177), ABCG2 (*p* = 0.526), Ki67 (*p* = 0.0987), and CK3 (*p* = 0.4000) in COMECs of different groups. RT-qPCR and western blotting/ELISA showed that COMECs of the LNC group expressed a significantly lower amount of basic fibroblast growth factor (bFGF) (*p* = 0.0038 for RT-qPCR, *p* = 0.0026 for western blotting) but more pigment epithelium-derived factor (PEDF) (*p* = 0.0172 for RT-qPCR, *p* = 0.0253 for western blotting) and soluble fms-like tyrosine kinase-1 (sFlt-1) (*p* < 0.0001 for RT-qPCR, *p* = 0.0064 for ELISA) than the COMECs of the 3T3 group. Furthermore, compared with COMECs of the 3T3 group, COMECs of the LNC group could reduce the viability (*p* = 0.0002) and tube formation (*p* = 0.0002) of HUVECs.

**Conclusions:**

LNCs could substitute 3T3 cells for expanding OMECs in vitro, and the COMECs obtained in this system are less likely to induce postsurgical neovascularization, which provides an alternative option for an ex vivo culture system and promotes the application of COMET.

**Electronic supplementary material:**

The online version of this article (10.1186/s11658-018-0133-x) contains supplementary material, which is available to authorized users.

## Background

Limbal stem cells (LSCs) play a vital role in maintaining a healthy ocular surface [1]. Significant damage to LSCs can lead to limbal stem cell deficiency (LSCD) characterized by pain, loss of vision, corneal neovascularization and conjunctivalization, chronic corneal inflammation, and subsequent scarring [2].

With improved stem cell technology, a variety of cell-based therapies have been suggested for LSCD to reconstruct the ocular surface [3]. Autologous cultivated limbal epithelial transplantation (CLET) has been reported as an effective and safe treatment [4, 5]. However, patients with bilateral LSCD have no cell source for CLET [6]. Therefore, autologous cultivated oral mucosal epithelial transplantation (COMET) was proposed to treat bilateral LSCD and achieved satisfactory results [7–10].

However, when 3T3 feeder cells were employed, varying degrees of neovascularization could be found in the peripheral cornea after COMET [9, 10]. Studies have demonstrated that compared with cultivated corneal epithelial cells, cultivated oral mucosal epithelial cells (COMECs) expressed fewer anti-angiogenic factors and more pro-angiogenic factors, in the presence of 3T3 feeder cells. This phenomenon may contribute to corneal neovascularization after COMET [11–13]. Additionally, it is considered that limbal stroma containing niche cells can regulate the LSCs [14]. As reported before [15], collagenase could successfully isolate LSCs and a subset of mesenchymal cells, i.e., limbal niche cells (LNCs), lying deep in the limbal stroma, and we have described a method to isolate and expand LNCs in vitro [16, 17]. Moreover, LNCs located in the limbal niche were proved to be able to support the growth of limbal epithelial cells [18, 19].

Thus, we wonder if allogenic LNCs could reduce the possibility of neovascularization after COMET. In this study, we cultivated oral mucosal epithelial cells (OMECs) with LNCs as reported not long ago [20], and assessed the differences between COMECs co-cultured with LNCs and COMECs co-cultured with 3T3 cells with respect to angiogenesis.

## Materials and methods

### Animals

Healthy Sprague-Dawley rats of either gender (weighing approximately 200 g) were purchased from the Laboratory Animal Center of Tongji Medical College of Huazhong University of Science and Technology (Wuhan, China). The animal-related activities were adherent to the Association for Research in Vision and Ophthalmology Statement for the Use of Animals in Ophthalmic and Vision Research. The rats were euthanized after anesthesia with pentobarbital sodium, then the biopsies were obtained. All experiments were approved by the Institutional Animal Care and Use Committee at Tongji Medical College, Huazhong University of Science and Technology (Permit Number: S641).

### Preparation of cells

Biopsies were rinsed three times with phosphate buffered saline (PBS; Hyclone, Logan, UT., USA) containing 50 μg/ml gentamicin (Invitrogen, Grand Island, NY, USA) and 1.25 μg/ml amphotericin B (Invitrogen, Grand Island, NY, USA). The cells were isolated as previously described [7, 16, 17], with some modifications. For allogenic LNCs, corneoscleral rims were prepared, cut into small segments, and digested with 1 mg/ml collagenase A (Roche, Indianapolis, IN, USA) at 37 °C for 3 h, and with 0.25% trypsin-EDTA solution (Gibco, Grand Island, NY, USA) at 37 °C for 15 min to generate single cells. Cell suspensions were seeded onto 6-well plates coated with 5% diluted Matrigel (60 μl/cm^2^) (Corning, MA, USA) in modified embryonic stem cell medium [16]. All experiments used LNCs of passage 3 to 5. 3T3 cells were purchased from the American Type Culture Collection and cultured directly on culture plates in DMEM/HIGH GLUCOSE (HyClone, South Logan, UT, USA) supplemented with 10% fetal bovine serum (Gibco, Thermo Fisher Scientific, Carlsbad, CA, USA) and 1% Penicillin-Streptomycin Solution (HyClone, South Logan, UT, USA). Oral biopsies were digested with 10 mg/ml Dispase II (Roche, Indianapolis, IN, USA) at 37 °C for 30 min. Subsequently, the OMEC sheets were peeled off, cut into small pieces, and seeded onto culture inserts with transparent polyethylene terephthalate membrane and small pores (with a diameter of 0.4 μm) (Corning, MA, USA) containing 50% diluted Matrigel (60 μl/cm^2^). The OMECs were co-cultured with confluent LNCs or 3T3 cells treated with mitomycin C (Sigma-Aldrich, St. Louis, MO, USA) in Keratinocyte Growth Medium 2 (KGM 2; PromoCell, Heidelberg, Germany) alone. When a confluent monolayer of COMECs was achieved, we lowered the KGM 2 level for airlifting for another week. Hematoxylin and eosin staining was performed to observe the morphological structures of COMEC sheets, normal oral mucosae, and normal corneas. Human umbilical vein endothelial cells (HUVECs) were purchased from Lonza (Hopkinton, MA, USA), cultured in Endothelial Cell Medium (ECM; ScienCell, CA, USA).

### Immunocytochemistry

After airlifting, the COMECs were treated with 10 mg/ml Dispase II at 37 °C for about 1 h, and 0.25% trypsin-EDTA solution at 37 °C for 10 min to obtain a single cell suspension. Then the single COMECs of different culture systems were transferred onto glass slides by cytospin (Shandon CytoSpin 3 Cytocentrifuge, Thermo Scientific, MA, USA) at 1200 g for 10 min at the density of 3–5 × 10^4^ single cells per slide to analyze protein expressions of p63α, ATP binding cassette subfamily G member 2 (ABCG2), Ki67, and cytokeratin 3 (CK3). For immunofluorescence staining, COMECs were fixed in 4% paraformaldehyde for 20 min at room temperature (RT), then washed with PBS. Except for the samples used to analyze ABCG2, the fixed cell samples were treated with 0.5% Triton X-100 for 20 min at RT. Then all samples were blocked with normal goat serum (AR1009, BOSTER, Wuhan, China) for 30 min at RT, after which the cells were sequentially incubated with specific primary antibodies (overnight at 4 °C) and corresponding secondary antibodies (1 h at 37 °C). Incubation with nonspecific normal mouse or rabbit immunoglobulin G (Beyotime Biotechnology, Shanghai, China) in place of primary antibodies was used as a negative control. LNCs of passage 3 were also investigated for markers of mesenchymal stem cells (Vimentin, N-cadherin, Oct4, and Sox2). Additionally, 4′,6-diamidino-2-phenylindole (DAPI; Dojindo, Tokyo, Japan) was used for nuclear counterstaining. Detailed information for the antibodies is listed in Table 1. For quantitation of p63α, ABCG2, Ki67, and CK3, we randomly selected three areas from one slide. More than 400 cells in total were analyzed for each cell marker. The percentages of cells positive for each marker were quantified by a masked researcher and representative images were captured with a fluorescence microscope (OLYMPUS BX53, Tokyo, Japan).

**Table 1:** Antibodies used for immunofluorescent staining

Antibodies	Sources	Dilution
p63α	BIOSS (BSM-33417 M)	1:100
ABCG2	BIOSS (BS-0662R)	1:100
Ki67	ABCAM (AB185627)	1 ěg/ml
CK3	BIOSS (BS-3646R)	1:100
Vimentin	BOSTER (BM0135)	1:100
N-cadherin	Proteintech (13769–1-AP)	1:100
Oct4	Proteintech (11263–1-AP)	1:100
Sox2	Proteintech (11064–1-AP)	1:100
Goat Anti-Mouse IgG/Cy3	BOSTER (BA1031)	1:100
Goat Anti-Rabbit IgG/Cy3	BOSTER (BA1032)	1:100

*ABCG2*: ATP binding cassette subfamily G member 2, *CK3*: cytokeratin 3

### RT-qPCR

Total RNA was isolated from COMECs by TRIzol reagent (Thermo Fisher Scientific, Carlsbad, CA, USA), and reverse transcribed to cDNA using the ReverTra Ace qPCR RT Kit (TOYOBO, Osaka, Japan) according to the manufacturer’s instructions. qPCR was performed with SYBR Green Master Mix (Bio-Rad, Hercules, CA, USA) to detect the mRNA expression of angiogenesis-related factors, including basic fibroblast growth factor (bFGF) [21], pigment epithelium-derived factor (PEDF) [22], soluble fms-like tyrosine kinase-1 (sFlt-1) [23], vascular endothelial growth factor (VEGF) [24], thrombospondin-1 (TSP-1) [25], tissue inhibitor of metalloproteinase-3 (TIMP-3) [26], and endostatin [27]. For detection of cDNAs, the following protocol was used on a QuantStudio 6 Flex Real-Time PCR System (Applied Biosystems, CA, USA): 2 min at 50 °C, 10 min at 95 °C for initial denaturation, 40 cycles of 30 s at 95 °C, and 30 s at 60 °C for primer annealing and extension. The primers are listed in Table 2. β-actin and glyceraldehyde 3-phosphate dehydrogenase (GAPDH) were used for normalization, and relative gene expression was analyzed by the 2^-ΔΔCt^ method [28].

**Table 2:** Primer sequences used for RT-qPCR

Name	Primer	Sequence	Size
β-actin	Forward	CACGATGGAGGGGCCGGACTCATC	240 bp
	Reverse	TAAAGACCTCTATGCCAACACAGT	
GAPDH	Forward	ACAGCAACAGGGTGGTGGAC	252 bp
	Reverse	TTTGAGGGTGCAGCGAACTT	
Rat bFGF	Forward	AGCATCACTTCGCTTCCCGC	226 bp
	Reverse	GGTTCGCACACACTCCCTTG	
Rat PEDF	Forward	GAAGGCGACGTTACCAACTC	242 bp
	Reverse	TCCGTGTCCCTCAGAACAAA	
Rat sFlt-1	Forward	CCCTCAGCCTACCATCAAGT	234 bp
	Reverse	GCCTTGCAGCTGTAGATTCC	
Rat VEGF	Forward	CGTCTACCAGCGCAGCTATTG	145 bp
	Reverse	CTCCAGGGCTTCATCATTGC	
Rat TSP-1	Forward	GTGTCAGTGGAGGAAGCTCT	193 bp
	Reverse	TGACATCTCCCTTTGCGACT	
Rat TIMP-3	Forward	GCCCTTTGGCACTCTGGTCT	151 bp
	Reverse	TGTCAGCAGGTACTGGTATTTGT	
Rat Endostatin	Forward	CCGTGCCCATCGTCAACCT	217 bp
	Reverse	GTCCGCCACGTCTCACAGTAG	

*GAPDH*: glyceraldehyde 3-phosphate dehydrogenase, *bFGF*: basic fibroblast growth factor, *PEDF*: pigment epithelium-derived factor, *sFlt-1*: soluble fms-like tyrosine kinase-1, *VEGF*: vascular endothelial growth factor, *TSP-1*: thrombospondin-1, *TIMP-3*: tissue inhibitor of metalloproteinase-3

### Western blot analysis

Proteins were extracted from COMECs using RIPA buffer (Beyotime Biotechnology, Shanghai, China) supplemented with phenylmethanesulfonyl fluoride (PMSF; Beyotime Biotechnology, Shanghai, China), and the protein concentration was quantified using a bicinchoninic acid protein assay kit (Beyotime Biotechnology, Shanghai, China). Then the lysates were denatured and proteins in each sample (40 μg) were separated with sodium dodecyl sulfate polyacrylamide gel electrophoresis. The protein bands were transferred to polyvinylidene fluoride (PVDF; Millipore, Darmstadt, Germany) membranes, followed by blocking with 5% skim milk, and incubated with specific primary antibodies and respective secondary antibodies (see Table 3), using GAPDH as a control. The immunoreactive proteins were detected with enhanced chemiluminescence (ECL; PerkinElmer, Waltham, MA, USA) and digitized with BandScan 5.0.

**Table 3:** Antibodies used in western blotting

Name	Sources	Dilution
Rabbit anti-GAPDH antibody	Goodhere (AB-P-R 001)	1:1000
Rabbit anti-bFGF antibody	Proteintech (11234–1-AP)	1:300
Rabbit anti-PEDF antibody	Bioworld (BS6690)	1:1000
Rabbit anti-VEGF antibody	Abcam (AB32152)	1:1000
Mouse anti-TSP-1 antibody	Santa Cruz (SC-73158)	1:50
Rabbit anti-TIMP-3 antibody	Proteintech (10858–1-AP)	1:1000
Rabbit anti-endostatin antibody	BIOSS (BS-0547R)	1:300
Goat anti-mouse IgG (peroxidase conjugate)	BOSTER (BA1051)	1:50000
Goat anti-rabbit IgG (peroxidase conjugate)	BOSTER (BA1054)	1:50000

*GAPDH*: glyceraldehyde 3-phosphate dehydrogenase, *bFGF:* basic fibroblast growth factor, *PEDF*: pigment epithelium-derived factor, *VEGF*: vascular endothelial growth factor, *TSP-1*: thrombospondin-1, *TIMP-3*: tissue inhibitor of metalloproteinase-3

### Enzyme linked immunosorbent assay (ELISA)

When the out-growth of OMECs covered approximately 90% of the insert membrane, the feeder cells were removed, and the culture medium was replaced with Basal Medium of KGM 2. After a 36-h incubation, the medium of different culture systems was collected and centrifuged at 2000 g for 10 min to remove cellular debris. The supernatant was recollected as conditioned medium (CM). Then, the concentrations of sFlt-1 in CM were determined by a commercial ELISA kit (Jiangsu Baolai Biotechnology, China), according to the manufacturer’s instructions with five replicates for each group including blank wells. Based on a standard curve, the sFlt-1 concentrations were determined. For standardization, the obtained values were normalized to cell numbers.

### Cell viability assay

The Cell Counting Kit-8 (CCK-8; Dojindo, Rockville, MD, USA) assay was conducted to determine the viability of HUVECs. After starvation of serum for 4 h, HUVECs were digested with trypsin-EDTA and divided into 3 groups according to the subsequent culture conditions. CM of COMECs was obtained as described above. HUVECs cultured in ECM were named the ECM group. HUVECs cultured in CM from COMECs co-cultured with LNCs or 3T3 cells were termed CM-LNCs or CM-3 T3, respectively. Two days later, the HUVECs of these 3 groups were resuspended and seeded onto 96-well plates (100 μl containing 5 × 10^4^ cells/well). After incubation at 37 °C for 24 h, 10 μL of CCK-8 reagent was added to each well, followed by a 4-h incubation. The optical density was measured at 450 nm using a Thermo Multiskan MK3 (Waltham, MA, USA).

### Tube formation assay

Each well of the 96-well plate was coated with 80 μl of Matrigel after incubation at 37 °C for 1 h. HUVECs were serum-starved for 4 h and reseeded onto the coated wells at a density of 1 × 10^4^ cells/well. The cells were divided into 3 groups as described for the cell viability assay. There were 5 wells in each group. Micrographs of tube formation were randomly captured using an OLYMPUS IX51 (Tokyo, Japan) at 8 h and day 1 after incubation at 37 °C. The branching points of tube-like structures at the end of day 1 were measured using ImageJ (National Institutes of Health, USA) by a masked observer. Three fields in each well were examined.

### Statistics

All assays were performed in triplicate independently with cells from different animals, and a minimum of three replicates were performed in each experiment. Data were analyzed and graphed using GraphPad Prism 7.0 software (San Diego, CA, USA). The Shapiro-Wilk test was performed to test the normality. After that, the normal data were presented as mean ± standard deviation, and the two-tailed unpaired Student’s t test was used to analyze the normal data of positive cell rate, RT-qPCR, and western blotting between two groups. When more than two groups were compared, the normal data were analyzed by one-way analysis of variance followed by Tukey’s post hoc test. Data that do not conform to a normal distribution were presented as median with interquartile range and analyzed by the Mann-Whitney U test. A *p* value of less than 0.05 was considered statistically significant.

## Results

### COMECs are obtained by co-culturing with LNCs or 3T3 cells

**Fig. 1:**
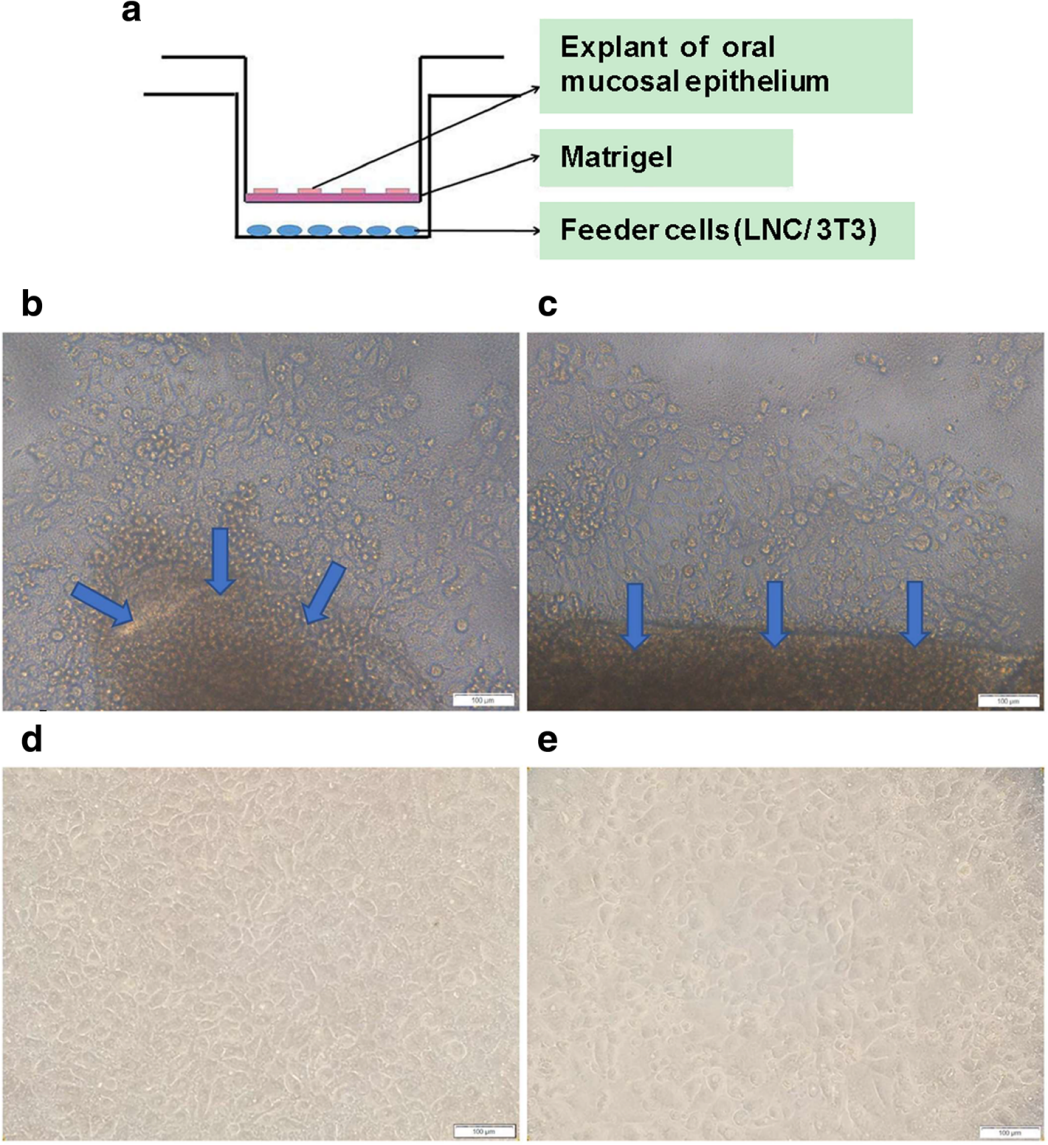
Morphological appearance of cultivated oral mucosal epithelial cells (COMECs) co-cultured with different feeder layers. **a** Schematic illustration of the culture model. COMECs co-cultured with LNCs (**b, d**) or 3 T3 cells (**c, e**). Epithelial cells migrated from the periphery of oral explants (blue arrows) on day 3 (**b, c**). A 90–100% confluent monolayer could be reached on day 9 (**d, e**). LNCs: limbal niche cells, scale bars: 100 μm

**Fig. 2:**
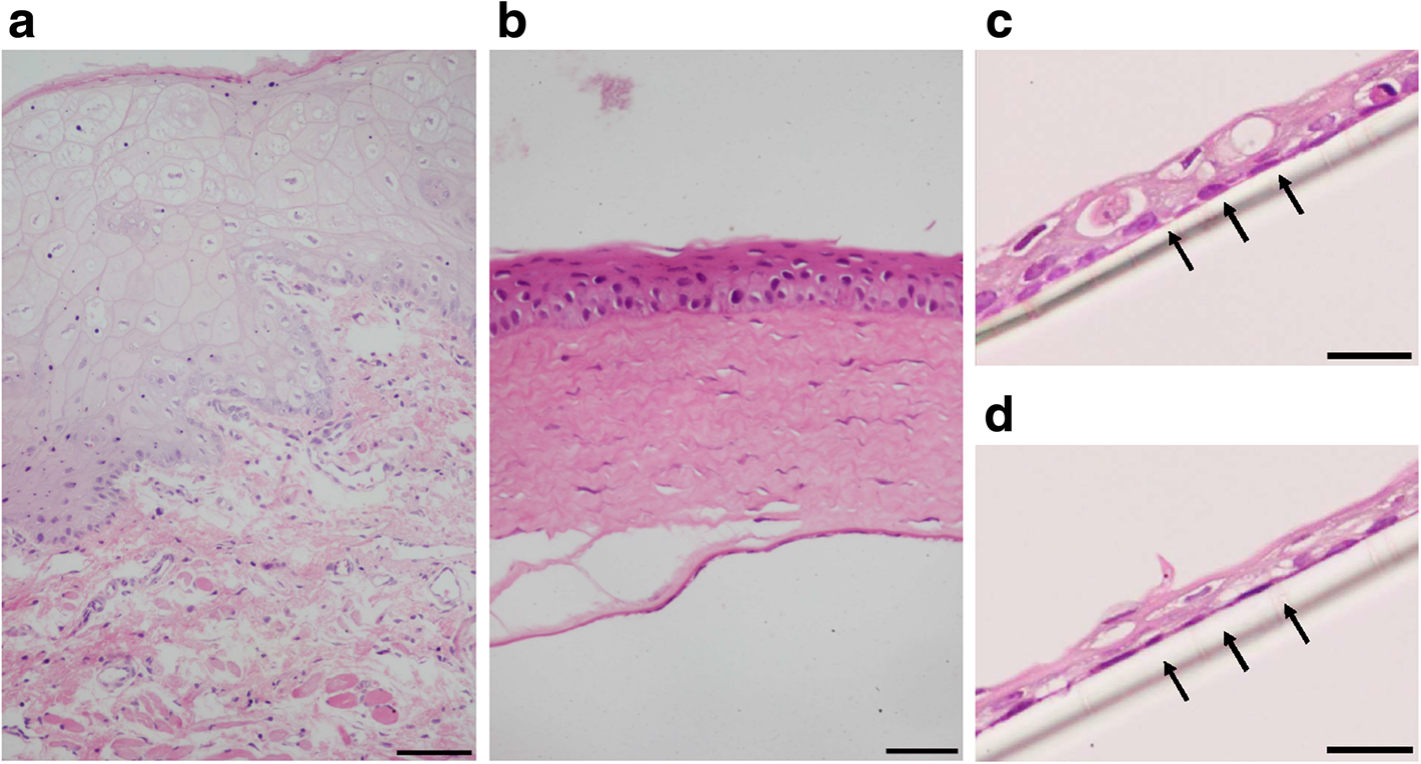
Representative images of hematoxylin and eosin staining. Stratified cultivated oral mucosal epithelial cell sheets co-cultured with LNCs (**c**) and 3 T3 cells (**d**) had 2–3 layers after airlifting for one week. These cell sheets resembled the normal corneal epithelial cells (**b**) rather than the native oral mucosal epithelial cells (**a**). Black arrows indicate the transparent polyethylene terephthalate membrane of culture insert. Scale bars: a: 100 μm, b: 50 μm, c and d: 25 μm

OMECs were expanded using the culture model described above (Fig. 1a). Microphotographs of COMECs in the LNC group (Fig. 1b and d) and the 3T3 group (Fig. 1c and e) were taken. The migrations of OMECs from oral explants were visible within 3 days (Fig. 1b and c). The cultures of different groups reached 90 to 100% confluence with a typical cobblestone or honeycomb pattern on day 9 (Fig. 1d and e). After one-week airlifting, stratified COMEC sheets were obtained in both culture systems (Fig. 2c and d). There was no obvious morphological difference between COMEC sheets cultured with LNCs and 3T3 cells. These sheets with small basal cells, flattened superficial cells, and 2–3 cell layers resembled normal corneal epithelial cells (Fig. 2b) more than the native oral mucosal epithelial cells (Fig. 2a).

### LNCs support the growth of OMECs

**Fig. 3:**
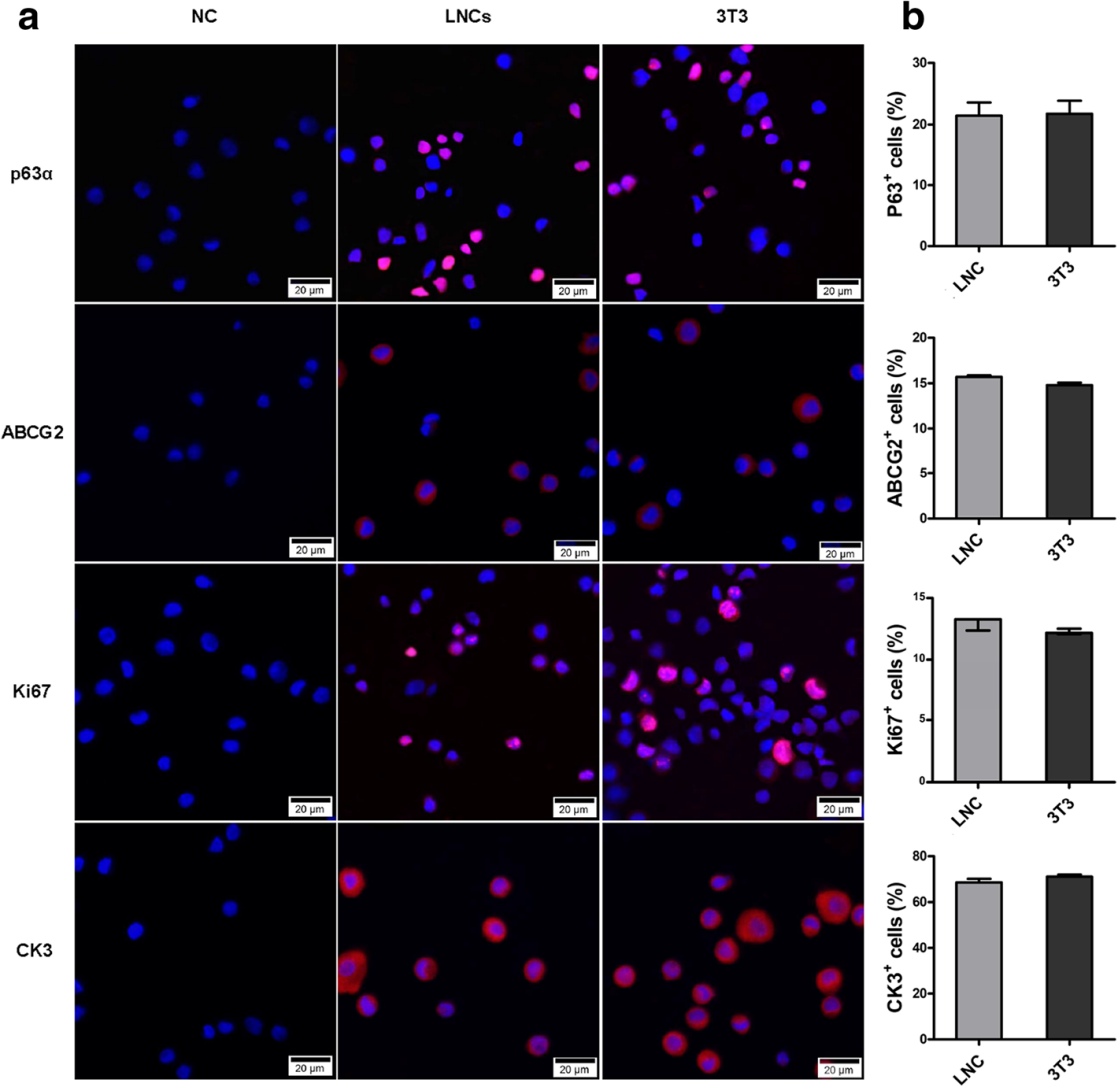
Identification and quantification of cell markers of cultivated oral mucosal epithelial cells (COMECs) co-cultured with LNCs and 3 T3 cells. **a** Representative images of p63α, ABCG2, Ki67, and CK3 in different culture systems. **b** The percentages of positive cells in different groups were approximately the same (*p* > 0.05). The results were expressed as mean ± standard deviation (p63α, ABCG2, and CK3) or median with interquartile range (Ki67). NC: negative control, LNCs: limbal niche cells, ABCG2: ATP binding cassette subfamily G member 2, CK3: cytokeratin 3, scale bars: 20 μm

**Fig. 4:**
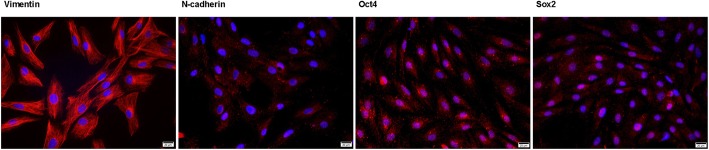
Molecular characterization of limbal niche cells (LNCs). LNCs of passage 3 stained positive for vimentin, N-cadherin, Oct4, and Sox2. Scale bars: 100 μm

To further investigate the characteristics of OMECs, we examined the expression of several cell markers by immunocytochemistry. Putative stem/progenitor cell markers, p63α [29] and ABCG2 [30], were detected in both groups (Fig. 3a). Further quantification analysis revealed no significant difference in the proportion of p63α^+^ or ABCG2^+^ cells between the two groups (*p* > 0.05) (Fig. 3b), which implied that the percentages of stem cells were similar in COMECs of different systems. We also examined Ki67 (Fig. 3a), a marker for active cell proliferation [31], and found that the percentages of Ki67^+^ cells in COMECs of different systems were approximately the same (*p* > 0.05) (Fig. 3b), indicating that the proliferation levels of COMECs in the two systems were equivalent. CK3 (Fig. 3a) is a marker of differentiated epithelial cells [7] and the immunofluorescence demonstrated no significant difference in the percentages of differentiated epithelial cells between the two culture systems (*p* > 0.05) (Fig. 3b). In addition, LNCs of passage 3 were vimentin^+^, N-cadherin^+^, Oct4^+^, and Sox2^+^, which means that the LNCs showed characteristics of mesenchymal stem cells (Fig. 4). Collectively, LNCs could maintain the stemness, proliferation, and differentiation of OMECs.

### Angiogenesis-related factors were differentially expressed by COMECs of different culture systems

As mentioned above, the imbalance of angiogenesis-related factors is the putative cause of postoperative neovascularization. Here, we compared the differences of the angiogenesis-related factors produced by COMECs of different culture systems.

**Fig. 5:**
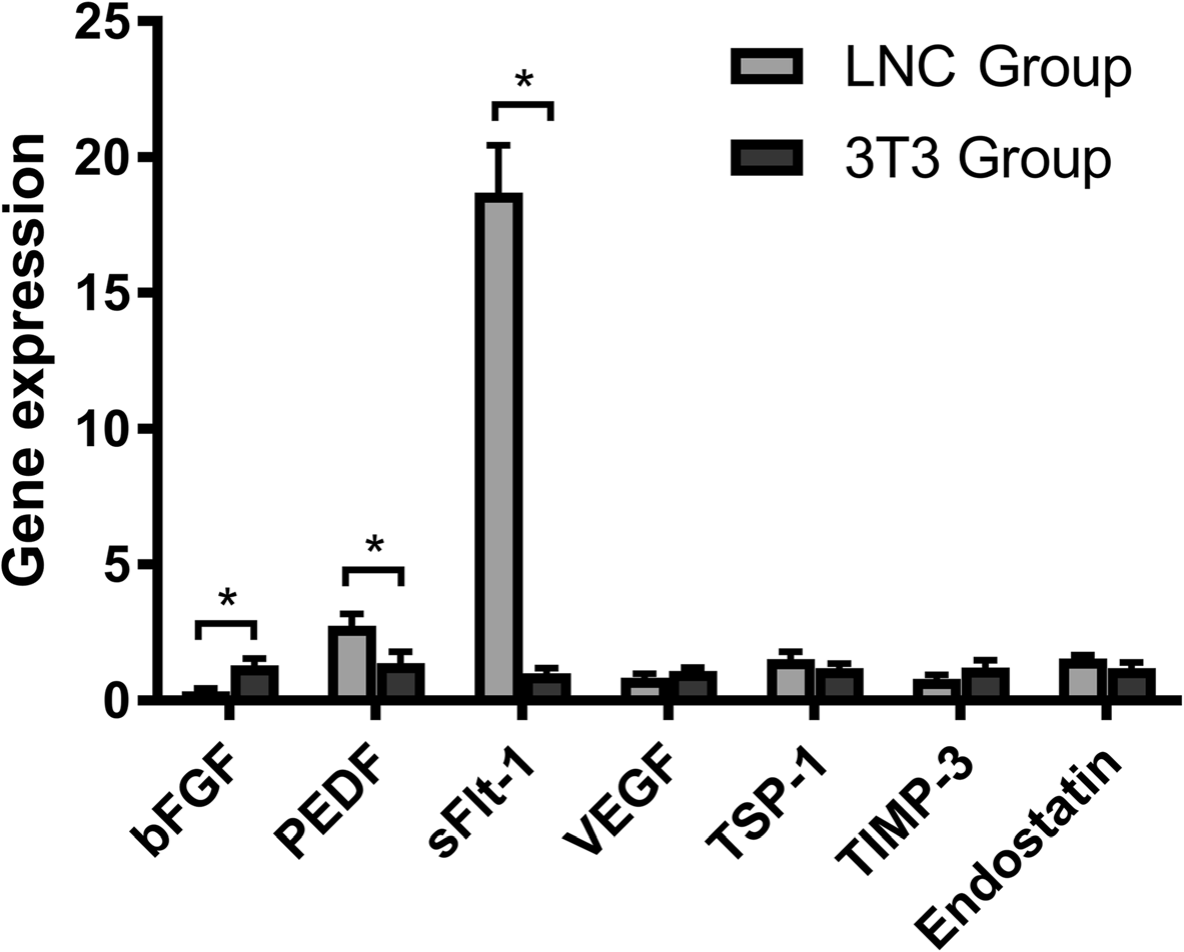
Relative mRNA expression of the angiogenesis-related factors of cultivated oral mucosal epithelial cells (COMECs) co-cultured with LNCs and 3 T3 cells. The relative genetic expression was investigated by RT-qPCR. β-actin and glyceraldehyde 3-phosphate dehydrogenase were used as internal controls. Data were expressed as mean ± standard deviation. LNCs: limbal niche cells, bFGF: basic fibroblast growth factor, PEDF: pigment epithelium-derived factor, sFlt-1: soluble fms-like tyrosine kinase-1, VEGF: vascular endothelial growth factor, TSP-1: thrombospondin-1, TIMP-3: tissue inhibitor of metalloproteinase-3, *: *p* < 0.05

**Fig. 6:**
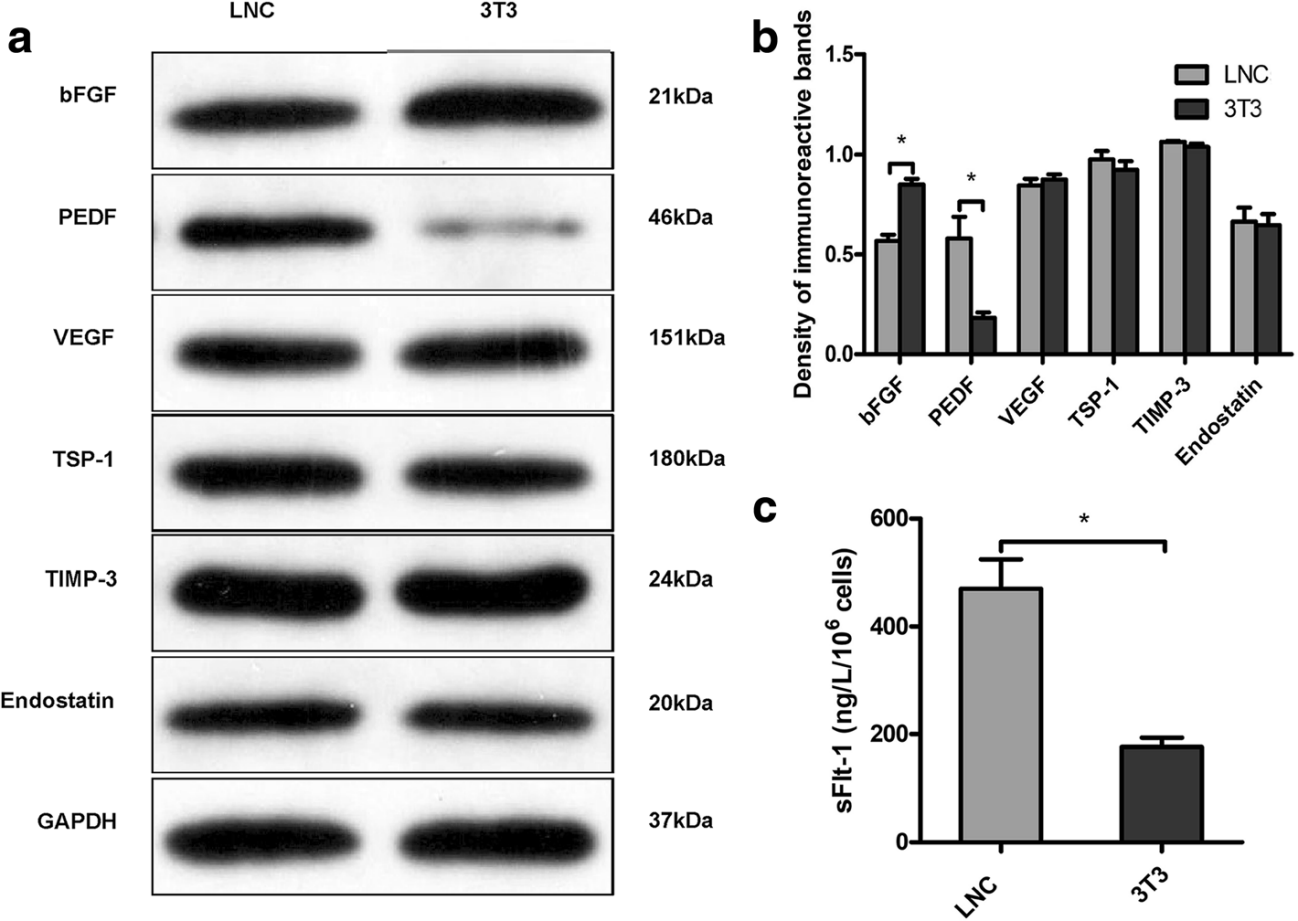
Expression of angiogenesis-related proteins of cultivated oral mucosal epithelial cells (COMECs) co-cultured with LNCs and 3 T3 cells. **a** Relative protein expression levels of bFGF, PEDF, VEGF, TSP-1, TIMP-3, and endostatin were detected by western blotting normalized with GAPDH. **b** Densitometric analysis of specific immunoreactive bands was performed. **c** The sFlt-1 concentrations in the conditioned medium were detected by enzyme-linked immunosorbent assays. The results were expressed as mean ± standard deviation. LNCs: limbal niche cells, bFGF: basic fibroblast growth factor, PEDF: pigment epithelium-derived factor, VEGF: vascular endothelial growth factor, TSP-1: thrombospondin-1, TIMP-3: tissue inhibitor of metalloproteinase-3, GAPDH: glyceraldehyde 3-phosphate dehydrogenase, sFlt-1: soluble fms-like tyrosine kinase-1, *: *p* < 0.05

The transcription levels of angiogenesis-related factors were measured using RT-qPCR (Fig. 5). Compared to the COMECs in the 3T3 group, the COMECs in the LNC group expressed less mRNA of the pro-angiogenic factor (bFGF [21]; *p* = 0.0038) but more mRNA of anti-angiogenic factors (PEDF [22] and sFlt-1 [23]; *p* = 0.0172 and *p* < 0.0001 respectively). To verify the gene expression levels, the protein levels of these factors were examined. Western blotting for bFGF, PEDF, VEGF, TSP-1, TIMP-3, and endostatin (Fig. 6a and b) and ELISA for sFlt-1 (Fig. 6c) reconfirmed that COMECs of the LNC group produced less bFGF (*p* = 0.0026), but more PEDF (*p* = 0.0253) and sFlt-1 (*p* = 0.0064) than the COMECs of the 3T3 group. The mRNA and protein expression levels of VEGF, TSP-1, TIMP-3, and endostatin of the COMECs in different groups showed no significant difference (*p* > 0.05; Fig. 5 and Fig. 6b). These results indicated that compared to the generally accepted 3T3 feeder cells, the use of LNC feeder cells could reduce the production of pro-angiogenic factors and increase the production of anti-angiogenic factors.

### COMECs in different culture systems possessed different angiogenic potential

**Fig. 7:**
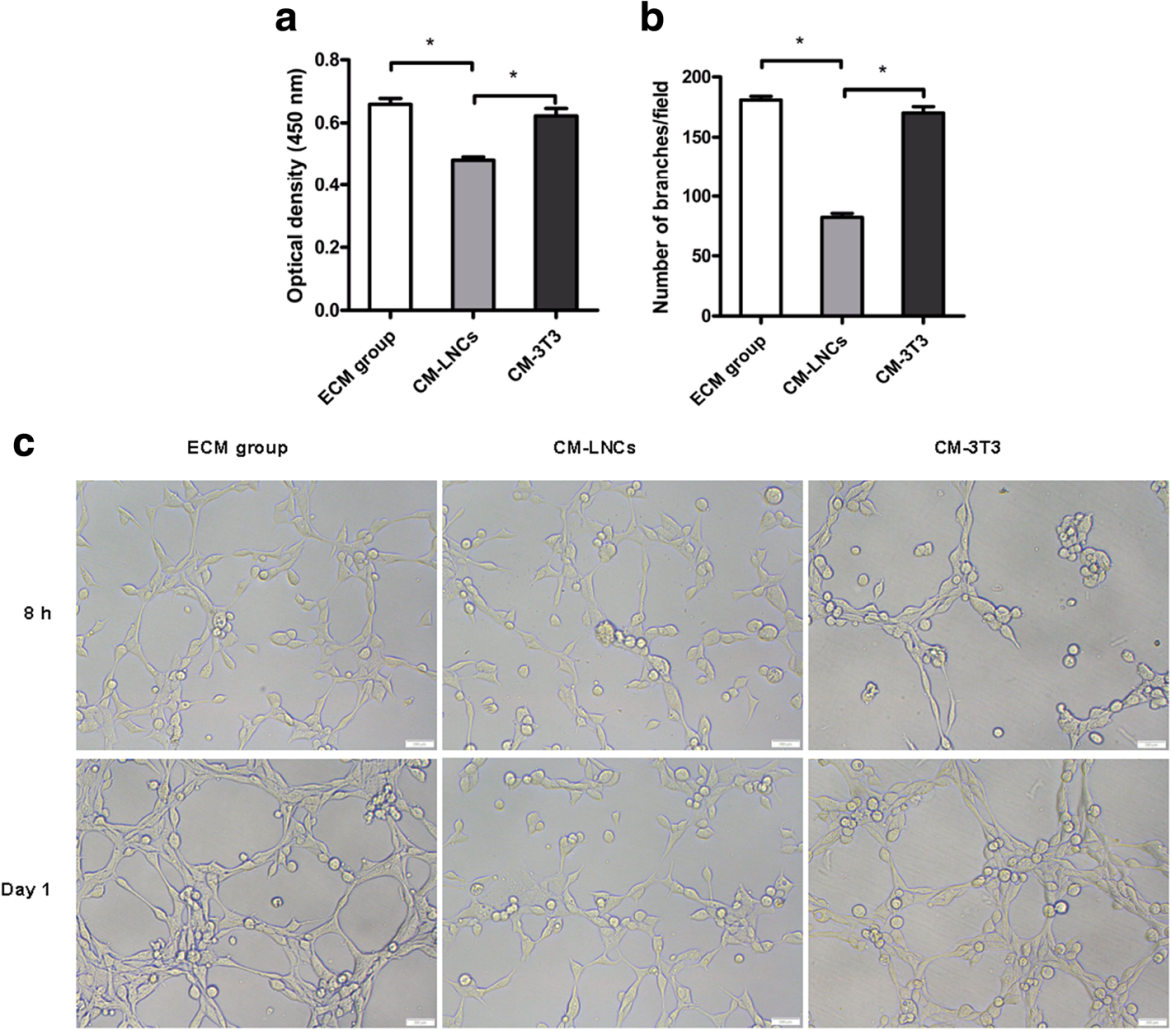
Cell viability assay and tube formation assay with human umbilical vein endothelial cells (HUVECs). HUVECs were cultured in conditioned medium of cultivated oral mucosal epithelial cells (COMECs) co-cultured with LNCs (CM-LNCs) and 3 T3 cells (CM-3T3s). Cell viability (**a**) and tube formation (**b, c**) of HUVECs were assessed. HUVECs cultured in Endothelial Cell Medium (ECM group) were used as control. **c** Micrographs of the tube-like structures in ECM group, CM-LNCs and CM-3T3s were captured. The results were expressed as mean ± standard deviation. LNCs: limbal niche cells, scale bars: 200 μm, *: *p* < 0.05

Knowing that COMECs obtained from the LNC group showed lower expression of pro-angiogenic factors but higher expression of anti-angiogenic factors, to further confirm the potential of LNCs to reduce angiogenesis, we measured the viability of HUVECs in different groups with CCK-8 assays. The results showed that the HUVECs of CM-LNCs had a significantly lower level of viability than the HUVECs of CM-3 T3 (*p* = 0.0002) and ECM groups (*p* < 0.0001) (Fig. 7a). In addition, we assessed the tube formation of HUVECs in different groups, and observed that the HUVECs of CM-LNCs formed significantly fewer tube-like structures than the HUVECs of CM-3 T3 (*p* < 0.0001) and ECM groups (*p* < 0.0001) (Fig. 7b). The viability and tube formation levels were not significantly different between the HUVECs of the ECM and CM-3 T3 groups (*p* > 0.05). These results illustrated that COMECs cultivated with LNCs had lower angiogenic potential than COMECs cultivated with 3T3 cells.

## Discussion

COMET, as a regenerative treatment for bilateral LSCD, often uses 3T3 cells for cultivating transplantable epithelial cell sheets [32]. Several groups have successfully obtained COMECs using feeder cells from other sources, such as dermal fibroblasts [33], mesenchymal stem cells derived from bone marrow [34], and conjunctiva fibroblasts [35]. Some studies have reported that OMECs can be expanded without feeder cells [36, 37]. However, none of these culture systems contained any component of the limbal niche, and the microenvironments may differ from the native limbal microenvironment. It has been believed that the stem cell niche, the specialized microenvironment surrounding stem cells (SCs), could play a critical role in the regulation of SCs [38, 39]. For the cornea, the limbal niche contains cells, and adjacent cellular and extracellular components [14, 19]. The cell types detected in the limbal niche include melanocytes, Langerhans cells, T-lymphocytes, and LNCs [18]. As a major constituent of the limbal niche, LNCs have been proved to possess the phenotypic characteristics of mesenchymal and embryonic SCs (reconfirmed by immunocytochemistry in the present study) and could support the self-renewal of limbal epithelial progenitor cells [15, 18]. Therefore, we used LNCs, as alternative feeder cells, to change the phenotype of COMECs by mimicking the native microenvironment of the limbus. Very recently, we successfully obtained corneal epithelial-like COMECs by using LNCs as feeder cells [20]. In the present study, we analyzed the markers for stemness, proliferation, and differentiation of COMECs in different groups and found that COMECs co-cultured with LNCs were not inferior to COMECs co-cultured with 3T3 cells as a resource for transplantation.

Postoperative corneal neovascularization is not uncommon after COMET [9, 10]. There is a delicate balance between pro-angiogenic and anti-angiogenic factors that determines corneal avascularity or lack thereof. Studies have shown that compared with cultivated corneal epithelial cells, COMECs co-cultured with 3T3 cells expressed fewer anti-angiogenic factors [11, 12], but more pro-angiogenic factors [13]. Moreover, some anti-angiogenic factors expressed in normal corneas could not be detected in corneal specimens from patients after COMET [40]. These molecular mechanisms may underlie postoperative neovascularization. It has been reported that bFGF could induce angiogenesis [21], PEDF was an inhibitor of angiogenesis [22], and sFlt-1 could inhibit angiogenesis and maintain corneal avascularity [23]. Our data showed that COMECs of the LNC group expressed lower levels of pro-angiogenic factors (bFGF) but higher levels of anti-angiogenic factors (PEDF, sFlt-1) than COMECs of the 3T3 group, which may imply that LNCs could reduce the likelihood of corneal neovascularization after COMET. In addition, COMECs cultured with LNCs had significantly lower capacity to maintain the viability of HUVECs and induce endothelial tube formation than COMECs cultured with 3T3 cells, indicating that COMECs co-cultured with LNCs were less capable of inducing angiogenesis, which further verified the role of LNCs in reducing postoperative neovascularization.

Previous studies have demonstrated that the different levels of biomarkers expressed by in vitro-cultivated cells were related to the feeder cells [19, 33, 34]. In the present study, different levels of angiogenesis-related factors were related to the different feeder cells used in the culture system. It has been acknowledged that SCs are regulated by particular microenvironments, and studies have reported the transdifferentiation of OMECs and hair follicle SCs into corneal epithelial-like cells in the limbal microenvironment [20, 41]. The microenvironment of COMECs co-cultured with LNCs has commonalities with the microenvironment of native LSCs. Thus, we speculated that the COMECs obtained in this culture system may respond to the microenvironment and present some phenotypes of the corneal epithelium, for example, low angiogenic potential [13].

## Conclusions

In summary, as capably as 3T3 cells, LNCs could maintain the stemness, proliferation, and differentiation of OMECs. Moreover, the COMECs obtained in this culture system had lower risk of inducing angiogenesis. Therefore, this culture system is theoretically superior to the commonly used system with 3T3 feeder cells, which may provide an alternative option for an ex vivo culture system and promote the application of COMET. Animal experiments are needed to reconfirm the effectiveness of LNCs, and the underlying mechanism needs to be clarified.

## Additional files

Additional file 1: Data of immunocytochemistry. (XLS 30 kb)

Additional file 2: Data of quantitative real-time RT-qPCR. (XLS 54 kb)

Additional file 3: Data of western blotting. (XLS 2913 kb)

Additional file 4: Data of ELISA. (XLS 58 kb)

Additional file 5: Data of CCK-8. (XLS 33 kb)

Additional file 6: Data of tube formation. (XLS 31 kb)

## Abbreviations

ABCG2: ATP binding cassette subfamily G member 2
bFGF: Basic fibroblast growth factor
CCK-8: Cell counting kit-8
CK3: Cytokeratin 3
CLET: Autologous cultivated limbal epithelial transplantation
CM: Conditioned medium
COMECs: Cultivated oral mucosal epithelial cells
COMET: Autologous cultivated oral mucosal epithelial transplantation
ECM: Endothelial cell medium
GAPDH: Glyceraldehyde 3-phosphate dehydrogenase
HUVECs: Human umbilical vein endothelial cells
KGM 2: Keratinocyte growth medium 2
LNCs: Limbal niche cells
LSCD: Limbal stem cell deficiency
LSCs: Limbal stem cells
OMECs: Oral mucosal epithelial cells
PEDF: Pigment epithelium-derived factor
RT: Room temperature
SCs: Stem cells
sFlt-1: Soluble fms-like tyrosine kinase-1
TIMP-3: Tissue inhibitor of metalloproteinase-3
TSP-1: Thrombospondin-1
VEGF: Vascular endothelial growth factor
